# Spatial Mapping of Genetic Liability to Psychiatric Disorders in the Adult Human Hippocampus

**DOI:** 10.1016/j.bpsgos.2026.100719

**Published:** 2026-03-05

**Authors:** Yusuf Baran, Darren Cameron, Adele Pryce-Roberts, Alexander L. Richards, Caleb Webber, Michael C. O’Donovan, Nicholas J. Bray

**Affiliations:** a Centre for Neuropsychiatric Genetics & Genomics, Division of Psychological Medicine & Clinical Neurosciences, Cardiff University, Cardiff, United Kingdom; b Betul-Ziya Eren Genome and Stem Cell Center, Erciyes Üniversitesi, Kayseri, Türkiye; c UK Dementia Research Institute at Cardiff, Cardiff University, Cardiff, United Kingdom

**Keywords:** Bipolar disorder, Depression, Genetic variants, GWAS, Hippocampus, Schizophrenia, Spatial transcriptomics

## Abstract

**Background:**

Common genetic variants associated with psychiatric disorders are enriched in genes with high expression specificity for hippocampal neurons. However, to date, these studies have been based on measures of gene expression from nuclei in liquid suspensions, where information on the precise location of cells within the assayed tissue is lost.

**Methods:**

We applied genetically informed spatial mapping of cells for complex traits (gsMAP) to test enrichment of common-variant genetic liability to schizophrenia, bipolar disorder, and major depressive disorder (MDD) in 13 hippocampal subregions according to expression specificity of associated genes using spatial transcriptomic (ST) data from 10 neurotypical adult donors. Then we used cellular deconvolution data from ST spots to perform further gsMAP analyses on cell populations within enriched subregions.

**Results:**

Compared with other hippocampal subregions, common-variant liability to schizophrenia, bipolar disorder, and, to a lesser extent, MDD, was significantly enriched in genes with higher expression specificity for the subiculum and neuron-rich areas of the cornu ammonis subfields and dentate gyrus. Within implicated regions, enrichments for schizophrenia and bipolar disorder were pronounced in ST spots containing glutamatergic neurons. Genes with higher expression specificity for the granule cell layer and subgranular zone of the dentate gyrus were more significantly enriched for association with bipolar disorder than for schizophrenia.

**Conclusions:**

Our findings support a role for glutamatergic neurons in all major subregions of the hippocampus in mediating common-variant liability to schizophrenia and bipolar disorder and provide evidence for the greater relative importance of the dentate gyrus in bipolar disorder.

## Plain Language Summary

Previous studies have provided evidence that cells of the hippocampus partially mediate genetic susceptibility to psychiatric disorders. Baran *et al.* test enrichment of common genetic variants associated with schizophrenia, bipolar disorder, and major depressive disorder in genes that are preferentially expressed in individual hippocampal subregions and cell types using recent spatial transcriptomic data from the adult human hippocampus. Their findings support a role for glutamatergic neurons in all major subregions of the hippocampus in mediating genetic susceptibility to schizophrenia and bipolar disorder and provide evidence for the greater relative importance of the dentate gyrus in bipolar disorder.

The hippocampus is a structure within the medial temporal lobe that serves an essential role in memory function (1). Reduced hippocampal volume has been observed in schizophrenia, bipolar disorder, and major depressive disorder (MDD) in structural neuroimaging meta-analyses (2, 3, 4). Involvement of the hippocampus in the pathophysiology of these conditions is also suggested by functional neuroimaging findings of abnormal hippocampal activation in these disorders (5, 6, 7). In schizophrenia, hippocampal dysfunction is further evidenced by neuropathological findings of reduced synaptic markers in the region (8,9).

It is possible to shed light on tissues of etiological relevance to complex traits by testing whether genes that are preferentially expressed in those tissues are enriched for genetic association with the trait (10,11). In the case of schizophrenia, bipolar disorder, and depression, common risk variants identified through genome-wide association studies (GWASs) are prominently enriched in genes that are preferentially expressed in the hippocampus and other brain regions serving higher cognitive functions (12, 13, 14, 15). Moreover, data from single-cell RNA sequencing/single-nuclei RNA sequencing (scRNA-seq/snRNA-seq) have enabled elucidation of the likely cellular mediators of common-variant liability to these disorders based on cell-specific gene expression (11, 12, 13, 14, 15, 16, 17, 18, 19, 20). These studies have implicated neurons of the hippocampus in mediating risk for all 3 psychiatric disorders (12,16, 17, 18, 19, 20). However, such data are derived from cells or nuclei in liquid suspensions, where information on the precise location of cells within the assayed tissue is lost. In addition, given difficulties in isolating whole cells from frozen postmortem tissue, enrichment analyses of human cell populations have so far been carried out using data only from isolated cell nuclei, which may not accurately reflect cytosolic and synaptic messenger RNA levels (16).

The recent availability of spatial transcriptomic (ST) datasets from the human brain provides the opportunity to test enrichment of common variability liability for psychiatric disorders based on gene expression from whole cells in their native tissue context. In ST studies, global RNA expression is assayed at individual locations (spots) within a tissue section. To date, the majority of ST data available from the human brain have been generated using the 10× Visium system, which measures gene expression in individual spots of 55 μm in diameter, spaced 100 μm apart. Here, we combine recent 10× Visium data from the adult human hippocampus (21) with large-scale GWAS data for schizophrenia (12), bipolar disorder (17), and MDD (18) to test enrichment of common-variant genetic liability for these conditions within genes with high expression specificity for individual hippocampal subregions and cell populations using analyses developed specifically for ST data (22).

## Methods and Materials

### Overview

**Figure 1:**
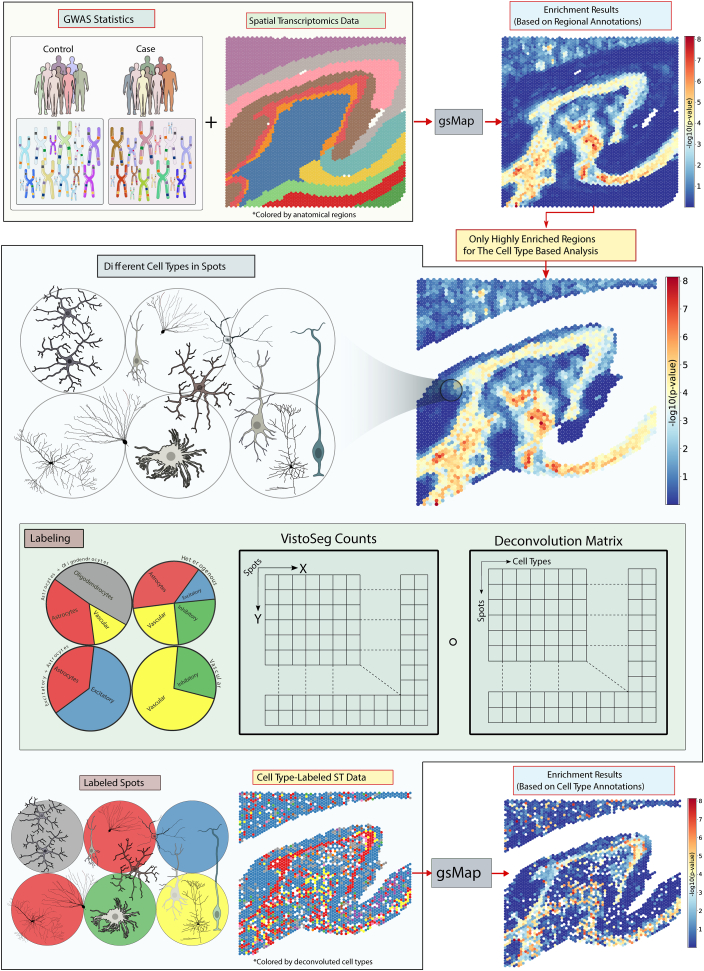
Graphical overview of this study. We obtained GWAS summary statistics for psychiatric disorders (12,17,18) and comparison traits (23, 24, 25, 26), along with ST data from the adult human hippocampus (21). We initially applied the gsMap method (22) to test enrichment of common genetic variants associated with these traits in genes with higher spatial expression specificity for each of 13 hippocampal subregions. Then we used cellular deconvolution and cell count data from the ST study (21) to label individual spots in subregions that were enriched for trait associations according to the cell types contained within them. Finally, we used gsMap to test for enrichment of common variants associated with each trait in genes with higher specificity for spots containing predicted cell types (or cell-type combinations) within each implicated subregion. gsMap, genetically informed spatial mapping of cells for complex traits; GWAS, genome-wide association study; ST, spatial transcriptomic.

A graphical overview of the methods and data used in this study is provided in Figure 1. We first used the genetically informed spatial mapping of cells for complex traits (gsMap) method (22) to test for enrichment of common-variant genetic liability to schizophrenia (12), bipolar disorder (17), and MDD (18) in hippocampal subregions according to spatial expression specificity of genes at associated loci using data from a recent ST study of the adult human hippocampus (21). Then we used cellular deconvolution and cell count data from the ST study to perform further gsMap enrichment analyses on cell populations within enriched hippocampal subregions.

### ST Data

**Figure 2:**
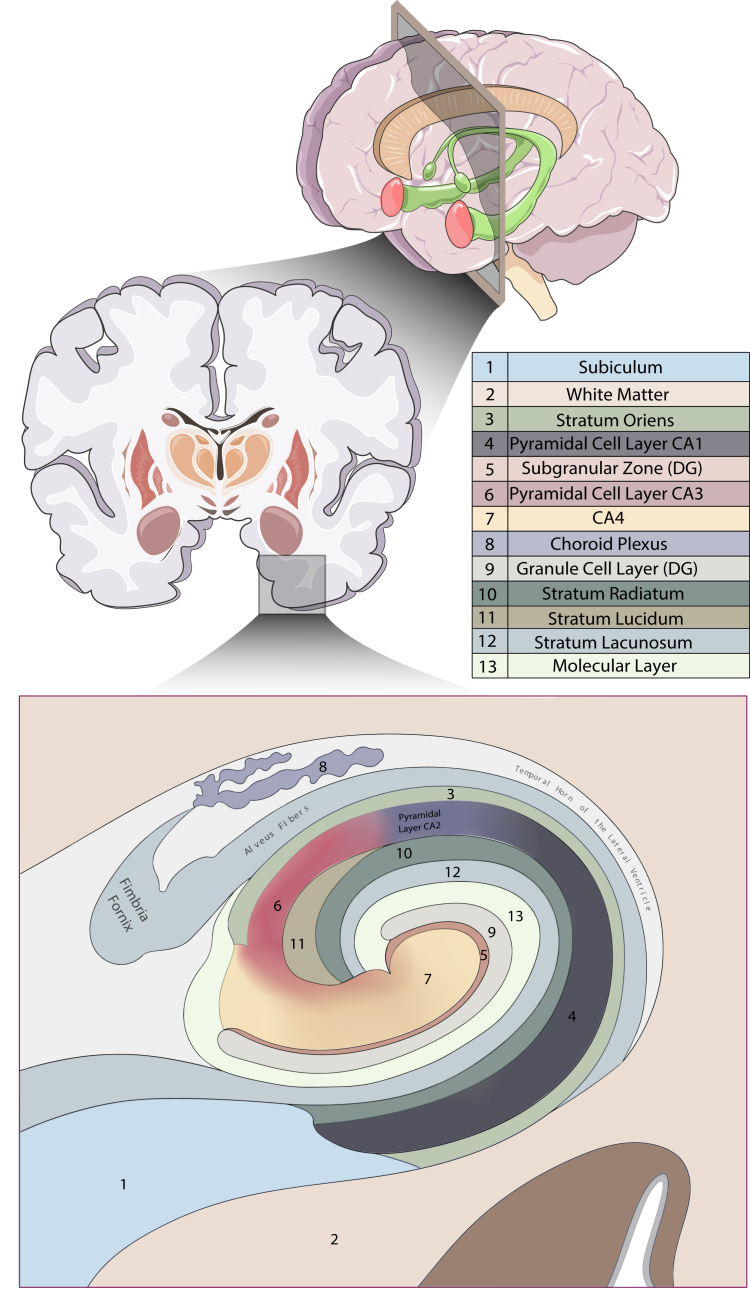
Sampling location and schematic of hippocampal subregions tested for enrichment of common-variant trait liability based on spatial gene expression specificity. Thompson *et al.* (21) generated spatial transcriptomic data from postmortem sections of the anterior human hippocampus from 10 neurotypical adult donors. The 13 hippocampal subregions defined by the authors and analyzed in this study are shown in the lower panel. CA*,* cornu ammonis; DG*,* dentate gyrus.

The hippocampal formation comprises several cytoarchitecturally distinct areas, including the dentate gyrus, the cornu ammonis (CA) subfields 1 to 4, the subicular complex, and, in some schema, the entorhinal cortex. The hippocampal ST data used in this study were obtained from the work of Thompson *et al.* (21), downloaded from the Gene Expression Omnibus (Accession Code: GSE264624). ST data were generated by the authors from postmortem sections of the anterior human hippocampus from 10 neurotypical adult donors using the 10× Genomics Visium Spatial Gene Expression platform. Between 2 and 5 capture areas were used for each donor, resulting in a total of 36 capture areas that collectively covered 13 histologically defined subregions of the hippocampal formation and surrounding tissue: the granule cell layer (GCL) and subgranular zone (SGZ) of the dentate gyrus, the CA1 pyramidal cell layer, the CA3 pyramidal cell layer (note: the pyramidal cell layer of the CA2 was not distinguished by the authors), the CA4 subfield, the subiculum, the stratum oriens, the stratum radiatum, the stratum lucidum, the stratum lacunosum-moleculare, the molecular layer, the choroid plexus, and white matter. The sampling location and 13 annotated regions are shown in Figure 2. Thompson *et al.* (21) used hematoxylin and eosin staining to verify cytoarchitecture across sections and, following quality control, retained 150,917 high-quality Visium spots for analysis. For a full description of samples and data generation, see the original publication (21). We filtered ST data to remove genes within the extended major histocompatibility complex region (hg19 coordinates: chr6 start = 25,000,000; end = 35,000,000) due to its extensive linkage disequilibrium (LD) and genes on the X or Y chromosomes, which could introduce sex-related donor bias (23). Then the processed data were converted into a .h5ad file format containing the count matrix, gene annotations, spot metadata, and spatial coordinates in compliance with gsMap input requirements.

### GWAS Data

GWAS summary statistics derived from the largest studies of schizophrenia (12), bipolar disorder (17), and MDD (18) were downloaded from the Psychiatric Genomics Consortium (PGC) website (https://pgc.unc.edu/for-researchers/download-results/). We restricted our analyses to European samples as LD score regression (LDSR) has been shown to be inappropriate for samples of mixed ancestry (24). As a comparison, we also tested GWAS summary statistics for a neurological disorder (genetic generalized epilepsy) (25), a neurodegenerative disease (Alzheimer’s disease) (26), intelligence (27), and the nonbrain phenotype of height (28).

A full list of GWAS datasets and weblinks is provided in Table S1. GWAS files were converted to sumstats format using the prepare_gwas function provided in the gsMap package. Gene coordinates were referenced using the Ensembl GRCh37 assembly (https://grch37.ensembl.org).

### Analysis Pipeline

Analyses of enrichment of common-variant liability to psychiatric disorders in relation to ST data were performed using gsMap (22), a package designed for ST-based stratified LDSR (S-LDSR) (29). We used default LD reference panels and genome version files provided by the gsMap package after verifying compatibility. The full pipeline was executed as described in the gsMap documentation (https://yanglab.westlake.edu.cn/gsmap/document/software).

To address technical noise and sparsity of gene detection in individual ST spots, gsMap trains a Graph Attention Network using spot gene expression profiles and spatial coordinates, resulting in the identification of homogeneous spots (microdomains). Gene specificity scores were computed for each gene in each spot by dividing the geometric mean of the gene’s expression ranking across the microdomain by the geometric mean of its expression ranking across all spots in the individual Visium capture area (or, for cell-type analyses, in the annotated hippocampal subregion within the capture area; see below). S-LDSR was then used to test whether genes with higher gene specificity scores in individual ST spots are enriched for single nucleotide polymorphism (SNP) heritability for the trait of interest. gsMap aggregated (one-tailed) enrichment *p* values for spots in each annotation (subregion or cell type) within each capture area using Cauchy combination tests (30). We considered enrichments significant when the median Cauchy *p* value for an annotation across all capture areas in which it was present was smaller than the Bonferroni-corrected threshold for the number of subregions/cell types tested in the analysis.

gsMAP analyses were first performed at the hippocampal subregion level, followed by analyses at the level of cell types within enriched subregions. Hippocampal subregion annotations were provided by the authors of the ST study (21). For the subregion analysis, we restricted our analysis to the 20 capture areas that covered at least half (i.e., 7) of the 13 subregions. Then we used cellular deconvolution results provided by Thompson *et al.* (21) to obtain cell-type annotations for individual spots within enriched subregions (see below).

### Cellular Deconvolution of ST Spots

Given that Visium spots are 55 μm in diameter, they typically capture 1 to 10 cells each, which could be of a single cell type or a mixture of cell types. To determine the cell types captured in individual spots, Thompson *et al.* applied cellular deconvolution methods based on snRNA-seq data they generated from adjacent hippocampal sections from the same 10 donors (21). To estimate cell-type proportions within individual spots in hippocampal subregions enriched for genetic liability to psychiatric disorders, we used the authors’ deconvolution results using robust cell-type decomposition (RCTD) (31), as they found that this method produced the most consistent performance on their hippocampal ST data. We combined these proportions with spotwise cell counts derived from the authors’ VistoSeg image processing tool (https://github.com/LieberInstitute/VistoSeg), available at their GitHub repository (32). By multiplying predicted cell proportions with total cell counts per spot, we estimated the number of cells per type contained in each spot.

For our cell-type analyses, we used the cell labels provided by Thompson *et al.* (21): “vascular cell,” “CSF,” “oligodendrocyte progenitor cell,” “oligodendrocyte,” “astrocyte,” “microglia/macrophages/T-cells,” “inhibitory (GABAergic) neuron,” and “excitatory (glutamatergic) neuron.” We labeled a spot as containing a single cell type if only cells of that type were present. If at least one cell of 2 different cell types (but no others) were present in the spot, we labeled the spot as both cell types. If 3 or more cell types were present in a spot, we labeled the spot as “heterogeneous” (or “excitatory + heterogeneous” if one of those cell types was an excitatory neuron).

## Results

### Enrichment of Common-Variant Liability to Psychiatric Disorders in Individual Subregions of the Adult Hippocampus

**Figure 3:**
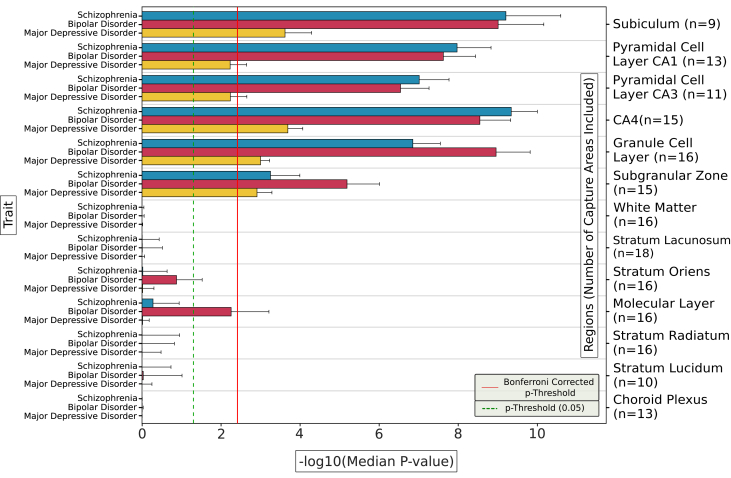
Enrichment of genetic associations with schizophrenia, bipolar disorder, and major depressive disorder in genes according to expression specificity across 13 subregions of the adult human hippocampus. Bars indicate the −log_10_ median of the Cauchy *p* values for enrichment of trait associations across all capture areas in which the subregion was included. Error bars indicate the standard error of the *p* values across capture regions containing the subregion. The number of capture areas including each subregion are provided in parentheses. The solid red line indicates the Bonferroni-corrected *p-*value threshold for the 13 tested subregions. CA*,* cornu ammonis.

We first used gsMAP to test for enrichment of common-variant liability to schizophrenia, bipolar disorder, and MDD in genes according to their expression specificity for the 13 hippocampal subregions annotated by Thompson *et al.* (21). Figure 3 shows the median genetic enrichment Cauchy *p* values obtained from between 9 and 18 capture areas per subregion. An example of gsMAP spot-level enrichments for schizophrenia in a single capture area is provided in Figure S1.

**Figure 4:**
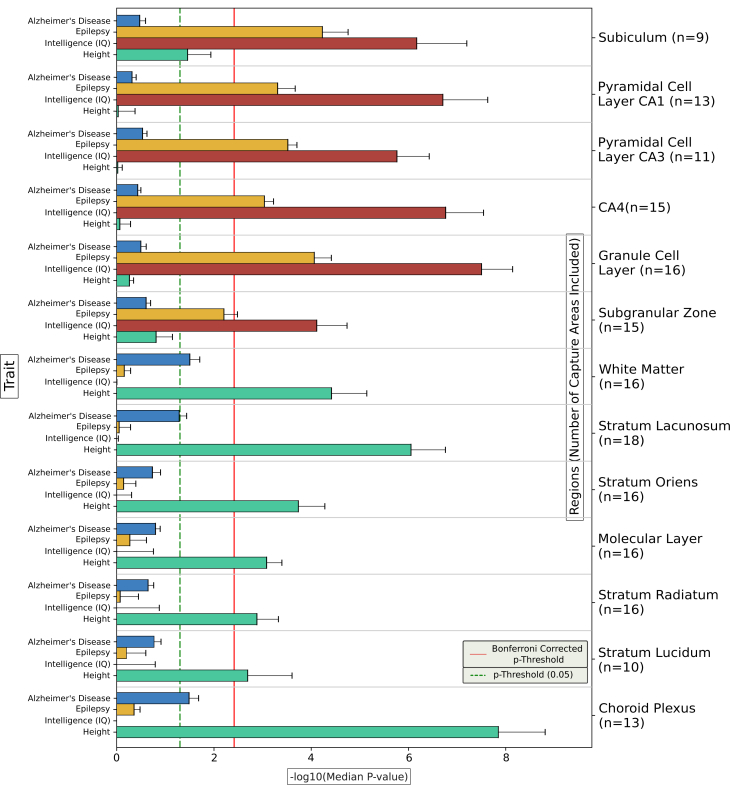
Enrichment of genetic associations with Alzheimer’s disease, genetic generalized epilepsy, intelligence, and height in genes according to expression specificity across 13 subregions of the adult human hippocampus. Bars indicate the −log_10_ median of the Cauchy *p* values for enrichment of trait associations across all capture areas in which the subregion was included. Error bars indicate the standard error of the *p* values across capture regions containing the subregion. The number of capture areas including each subregion are provided in parentheses. The solid red line indicates the Bonferroni-corrected *p*-value threshold for the 13 tested subregions. CA*,* cornu ammonis.

Common variants conferring risk for all 3 psychiatric disorders were significantly enriched in genes with high expression specificity for the GCL and SGZ of the dentate gyrus, the CA4 subfield, and the subiculum. Common-variant liability to schizophrenia and bipolar disorder was also prominently enriched in genes with high expression specificity for the pyramidal cell layers of the CA1 and CA3 subfields. The significance of enrichments was substantially lower for MDD compared with schizophrenia and bipolar disorder in all 6 of these subregions. While median enrichment *p* values were similar in schizophrenia and bipolar disorder for most of these subregions, they were notably smaller in the GCL and SGZ of the dentate gyrus for bipolar disorder; we confirmed smaller individual Cauchy enrichment *p* values for bipolar disorder compared with schizophrenia in these 2 subregions using Wilcoxon rank-sum tests (GCL *p* = .00003; SGZ *p* = .003) (Figure S2). To identify genes that potentially explain these differences, we used the MAGMA SNP-wise mean model (33) to generate gene-level association *p* values for bipolar disorder and schizophrenia for the 1000 genes with the highest mean gene specificity scores for the GCL and for the SGZ across all capture areas (Tables S2 and S3). Genes with notably smaller MAGMA *p* values for bipolar disorder than schizophrenia that have high expression specificity for these subregions include *SPTBN2* in the GCL (bipolar disorder *p* = 3.1 × 10^−8^; schizophrenia *p* = .0188) and *FADS2* in the SGZ (bipolar disorder *p* = 1.8 × 10^−12^; schizophrenia *p* = .009). As a comparison to the psychiatric phenotypes, we tested enrichment of common variants associated with a neurodegenerative disease (Alzheimer’s disease), a neurological disorder (genetic generalized epilepsy), cognition (intelligence) and the nonbrain phenotype of height according to gene expression specificity for the 13 hippocampal subregions (Figure 4). Common variants associated with Alzheimer’s disease were not enriched in genes with high expression specificity for any hippocampal subregion, showing nominally significant enrichments (uncorrected *p* < .05) only in the choroid plexus and white matter. Common variants associated with epilepsy and intelligence were, like those associated with psychiatric disorders, significantly enriched in genes with higher expression specificity for the subiculum, dentate gyrus, and CA subfields. Variants associated with height were not enriched in genes with preferential expression for any of the hippocampal subregions implicated in psychiatric disorders; however, they were significantly enriched in genes with higher expression specificity for other subregions, particularly the highly vascularized choroid plexus. To shed light on biological processes that might mediate enrichment of genetic liability to neuropsychiatric disorders in subregions of the hippocampus, we 1) generated gene-level MAGMA association *p* values for all 3 disorders for the 1000 genes with the highest mean gene specificity scores for each implicated subregion across capture areas, and 2) performed Gene Ontology (GO) analysis on all genes with MAGMA association *p* values < 5 × 10^−5^ using g:Profiler (34), with all expressed genes as the background for each subregion. Genes with high specificity for implicated subregions that are associated with either schizophrenia, MDD, or intelligence were prominently enriched for terms relating to the synapse, although the genes driving these enrichments in the 3 traits only partially overlapped (Table S4).

### Enrichment of Common-Variant Genetic Liability to Psychiatric Disorders in Spatially Defined Cell Populations of the Adult Hippocampus

**Figure 5:**
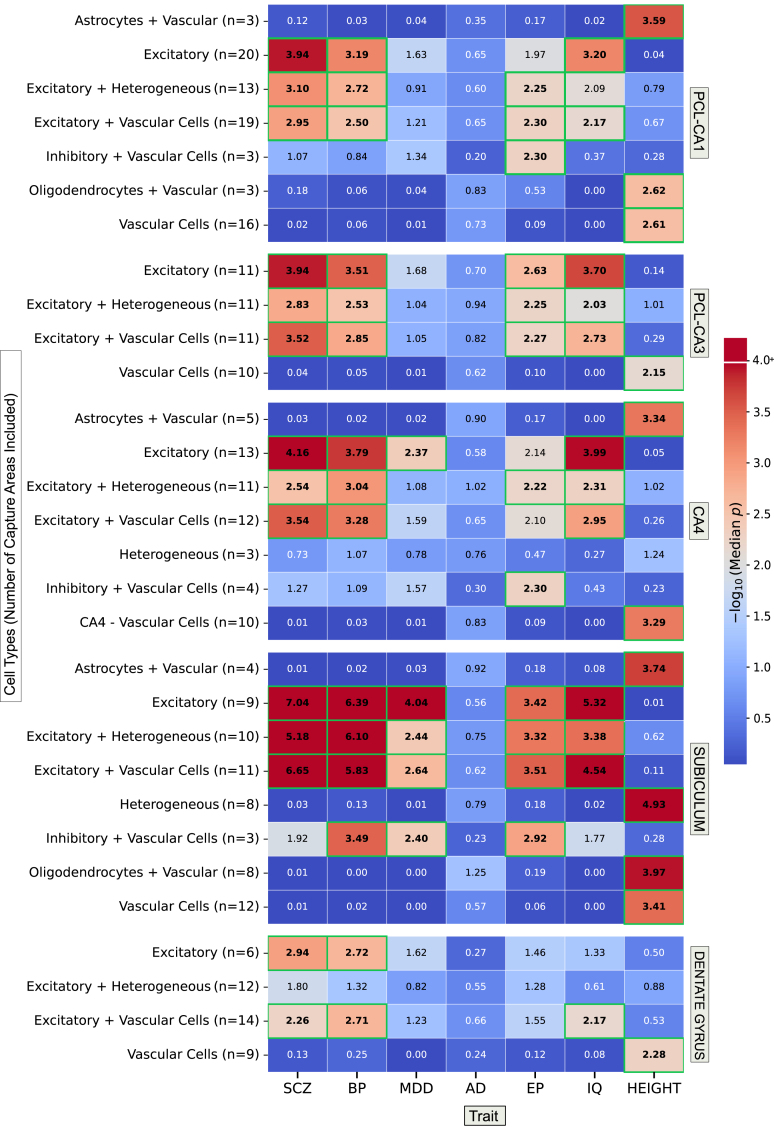
Enrichment of genetic associations with psychiatric disorders and comparison traits in genes according to expression specificity in spots containing different cell types. The heatmap shows the −log_10_ median of the Cauchy *p* values for enrichment of trait associations in each cell type in hippocampal subregions implicated in psychiatric disorders across all capture areas. The number of capture areas for each subregion that included spots with the predicted cell type(s) are provided in parentheses. To maximize the number of spots containing each cell type, we combined those belonging to the granule cell layer and subgranular zone. Data for each subregion are also shown as bar charts with error bars to indicate variability in Cauchy *p* values across capture areas (Figures S3–S8). AD*,* Alzheimer’s disease; BP*,* bipolar disorder; EP*,* genetic generalized epilepsy; MDD*,* major depressive disorder; PCL*,* pyramidal cell layer; SCZ*,* schizophrenia.

To determine spatially defined cell populations of the adult hippocampus that are likely to mediate genetic liability to psychiatric disorders, we next combined gsMap with (RCTD-based) cellular deconvolution of individual ST spots within the hippocampal subregions that were found to be enriched for genetic association with the conditions (Figure 5 and Figures S3–S5). Common-variant liability to schizophrenia and bipolar disorder was prominently enriched in genes with higher expression specificity for spots containing excitatory (glutamatergic) neurons compared with spots containing other cell types in all tested CA subfields, the dentate gyrus, and the subiculum. Common variants associated with MDD were enriched in genes with higher expression specificity for spots containing excitatory (glutamatergic) neurons in the dentate gyrus, subiculum, and CA4 subfield only. Within the subiculum, common-variant liability to bipolar disorder and MDD was also significantly enriched in genes with higher expression specificity for spots predicted to contain both inhibitory (GABAergic [gamma-aminobutyric acidergic]) neurons and vascular cells.

As a comparison, we also used gsMap to test enrichment of common variants associated with Alzheimer’s disease, intelligence, genetic generalized epilepsy, and height in genes with expression specificity for ST spots containing different types within the same hippocampal subregions (Figure 5 and Figures S6–S8). As with common genetic variation associated with schizophrenia and bipolar disorder, genetic variation associated with intelligence was prominently enriched in genes with high expression specificity for spots predicted to contain excitatory (glutamatergic) neurons. Like that for bipolar disorder and MDD, common-variant liability to epilepsy was enriched in genes with high expression specificity for spots predicted to contain excitatory (glutamatergic) neurons or both inhibitory (GABAergic) neurons and vascular cells in the subiculum. In contrast to the 3 tested psychiatric disorders, common variants associated with epilepsy were also enriched in genes with high expression specificity for spots containing both inhibitory (GABAergic) neurons and vascular cells in the CA1 and CA4 subfields. Common variants associated with Alzheimer’s disease were not significantly enriched in genes with higher expression specificity for spots containing any particular cell types, while those associated with height were significantly enriched in genes with higher expression specificity for spots containing vascular cells.

### Genes Containing an Excess of Rare Damaging Coding Variants in Schizophrenia Have Higher Expression Specificity for the CA1 and CA4 Subfields and GCL in the Hippocampus

**Figure 6:**
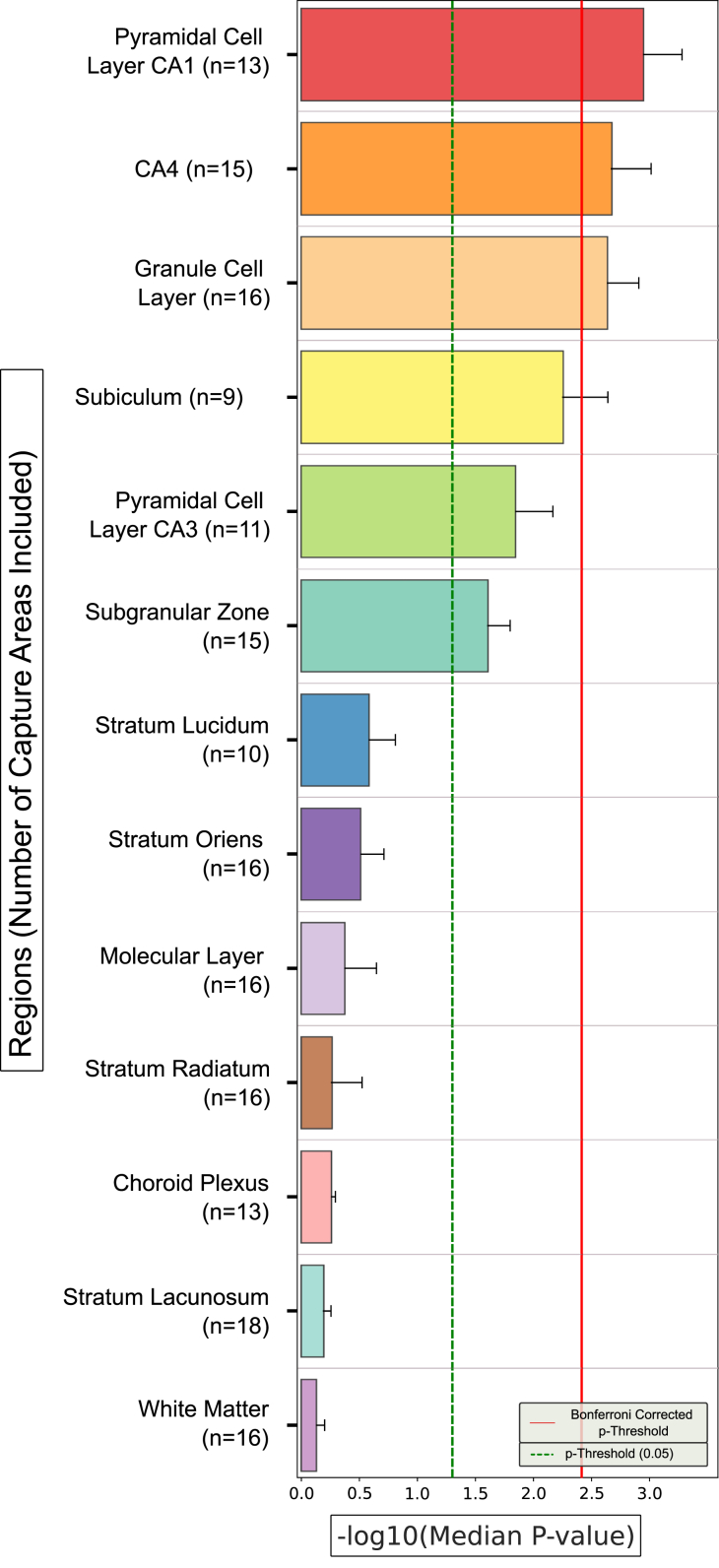
Higher expression specificity of genes containing an excess of damaging rare coding variants in schizophrenia in particular subfields of the hippocampus. Bars indicate the −log_10_ median *p* values for the one-sided Wilcoxon rank-sum tests performed on each capture area comparing the expression specificity rankings of 27 genes identified by Chick *et al.* (35) as containing an excess of rare damaging coding variants against the rankings of all other genes they tested. The number of capture areas including each subregion (and therefore tests performed for that subregion) are provided in parentheses. Error bars indicate the standard error of the *p* values across those capture areas. The solid red line indicates the Bonferroni-corrected *p*-value threshold for the 13 tested subregions. CA*,* cornu ammonis.

Although gsMAP is designed to test enrichment of common-variant genetic associations in ST data, we were able to use the mean gene expression specificity scores in each hippocampal subregion within individual capture areas to test whether genes that have been found to contain an excess of rare damaging coding variation in schizophrenia (35) have higher expression specificity for any subregion. One-sided Wilcoxon rank-sum tests comparing the expression specificity rankings of 27 rare-variant schizophrenia genes identified by Chick *et al.* (35) at false discovery rate–corrected *p* < .05 against the rankings of all other genes that they tested indicated higher specificity of the former in the pyramidal cell layer of the CA1 subfield, the GCL of the dentate gyrus, and the CA4 subfield (Figure 6).

## Discussion

The hippocampus has been implicated in the pathophysiology of psychiatric disorders through neuroimaging studies (2, 3, 4, 5, 6, 7), neuropathological investigations (8,9), and expression specificity of genes associated with these conditions at the neuroanatomical (12, 13, 14) and cellular (12,16, 17, 18, 19, 20) level. Here, we expand upon these findings by investigating expression specificity of genes associated with these disorders at the level of hippocampal subregions and the cell types within them. Compared with other hippocampal subregions, common-variant liability to psychiatric disorders was found to be enriched in genes with higher expression specificity for the subiculum and neuron-rich areas of the CA subfields and dentate gyrus. The pattern of enrichments for MDD was similar to those observed for schizophrenia and bipolar disorder but did not reach the same level of statistical significance. It is possible that this finding to some extent reflects the lower SNP heritability of MDD compared with the other 2 disorders (15), although this would be at least partially mitigated by the considerably larger sample size of the MDD GWAS utilized (Table S1). A difference between schizophrenia and bipolar disorder in the significance of regional enrichments was observed in the GCL and SGZ of the dentate gyrus, with genes with high expression specificity for these subregions more significantly enriched for associations with bipolar disorder. Using cellular deconvolution of ST spots, we found particularly strong enrichment of genetic liability to schizophrenia and bipolar disorder in genes with high expression specificity for glutamatergic neurons within implicated hippocampal subfields.

Our hippocampal subregion results corroborate, in humans, the findings of Song *et al.* who reported strong enrichment of schizophrenia common-variant liability in genes with high expression specificity for the subiculum, dentate gyrus, CA1, and CA3 subregions using ST data from the adult mouse brain (22). In that study, common-variant associations with depression were also found to be enriched in these hippocampal subregions, although, consistent with our findings, these enrichments were less significant than for schizophrenia (being stronger in the midbrain) (22). Our use of data from a recent large GWAS of bipolar disorder (17) revealed enrichment of common-variant genetic liability at similar levels of significance to schizophrenia in most hippocampal subregions. A notable disparity was observed in genes with higher expression specificity for the GCL and SGZ of the dentate gyrus, where enrichments were more significant for bipolar disorder. This is consistent with a recent study in which common genetic variation differentiating bipolar disorder from schizophrenia was found to be enriched in genes with high expression specificity for cells annotated as dentate gyrus neurons in cell-suspension snRNA-seq data (36). Together, these findings suggest that the dentate gyrus plays a more important role in the pathophysiology of bipolar disorder than schizophrenia. Unlike other subregions of the hippocampus, the dentate gyrus contains granule cells as the primary glutamatergic neuron. It has been reported that induced pluripotent stem cell–derived granule neurons from people with bipolar disorder show a hyperexcitable phenotype (37).

The genetic enrichments we observed following cellular deconvolution of hippocampal ST spots provide neuroanatomical support for earlier findings, based on cell-suspension scRNA-seq/snRNA-seq data, that implicate glutamatergic neurons from various subregions of the adult hippocampus in genetic risk for psychiatric disorders (12,16,17,19). In those studies, genetic liability to psychiatric disorders was found to be enriched in genes with high expression specificity for pyramidal neurons of the CA1 to CA3 subfields (12,16,17,19), granule neurons of the dentate gyrus (12,16,17,19), and mossy cells of the CA4 subfield (19), which were annotated on the basis of cellular gene expression markers. In addition to supporting these findings using ST data from intact human hippocampal tissue, we implicate both glutamatergic and GABAergic neurons of the subiculum in the genetic pathophysiology of these conditions. These latter enrichments are likely to reflect risk variants operating in glutamatergic pyramidal cells and parvalbumin-positive GABAergic interneurons, which are known to be the major excitatory and inhibitory neurons in this brain region. The subiculum is the major output structure of the hippocampus, sending projections to various cortical and subcortical regions, including the entorhinal cortex, medial prefrontal cortex, nucleus accumbens, amygdala, and thalamus (38). Although the subiculum was found by Song *et al.* (22) to be a brain region showing pronounced enrichment of genetic liability to psychiatric disorders, we are not aware of any previous studies implicating specific cell types within this structure in the genetic pathophysiology of these conditions.

We compared the pattern of enrichments of genetic liability to psychiatric disorders with those for variants associated with intelligence, Alzheimer’s disease, genetic generalized epilepsy, and the nonbrain phenotype of height. Our findings for intelligence are largely in keeping with those of Song *et al.* (22), who found strong enrichment of variants associated with IQ in genes with high expression specificity for subregions of the hippocampus using adult mouse brain ST data. Although we found that variants associated with intelligence are, like those associated with the tested psychiatric disorders, enriched in genes with high specificity for hippocampal glutamatergic neurons and in GO terms related to the synapse, the genes driving these enrichments only partially overlap (Table S4), consistent with the modest (negative) genetic correlation between intelligence and psychiatric disorders (26). Therefore, it is plausible that genetic variants operating through the hippocampus to increase risk for these disorders partially do so through effects on hippocampal processes other than those involved in general intelligence. We observed no enrichment of variants associated with Alzheimer’s disease in genes with high expression specificity for hippocampal subregions or in ST spots predicted to contain neurons. This is consistent with the findings of Song *et al.* (22) using adult mouse brain ST data, as well as findings of studies using human brain snRNA-seq data of enrichment of variants associated with Alzheimer’s disease in genes with high specificity for microglia (16,39). Unlike our findings for psychiatric disorders, common variants associated with genetic generalized epilepsy were found to be enriched in genes with high specificity for ST spots of the CA1 and CA4 subfields that were predicted to contain GABAergic neurons, in addition to spots predicted to contain glutamatergic neurons. These findings are consistent with previous analyses that have shown enrichment of variants associated with epilepsy in genes with high expression specificity for hippocampal tissue and both GABAergic and glutamatergic neurons in the brain (24). As expected, we observed no enrichment of variants associated with height in genes with high expression specificity for subregions of the hippocampus that contained a concentration of neurons, instead finding enrichments in spots predicted to contain vascular cells.

Current ST datasets from the adult human brain are limited, making comparison with other brain regions difficult. However, using ST data from the adult mouse brain, Song *et al.* (22) found that only genes with high expression specificity for the cerebral cortex were more significantly enriched for genetic liability to schizophrenia than in those with high specificity for subregions of the hippocampal formation. Future ST datasets, encompassing multiple human brain regions, will help clarify the relative importance of hippocampal areas in mediating genetic risk to psychiatric conditions. In addition, ST datasets from the adult human brain have generally used technology that does not attain single-cell resolution. To address this limitation, we used cellular deconvolution data to predict cell types within individual spots. However, higher resolution ST technology is now available and is beginning to be applied to human brain (40). These data are likely to be important in refining our understanding of the cellular underpinnings of psychiatric disorders, including differences between them.

## Supplementary Material

Tables S1–S4

Key Resources Table
