# Supplementary material for: Spatial Mapping of Genetic Liability to Psychiatric Disorders in the Adult Human Hippocampus
Source: Biol Psychiatry Glob Open Sci. 2026 Mar 5;6(3):100719. doi: 10.1016/j.bpsgos.2026.100719 (PMC13094429; doi:10.1016/j.bpsgos.2026.100719)
Supplement: Figure S1–S8 [file mmc1.pdf]

SUPPLEMENTARY INFORMATION

Spatial Mapping of Genetic Liability to Psychiatric Disorders in the Adult Human Hippocampus

Baran *et al.*

Supplementary Figures

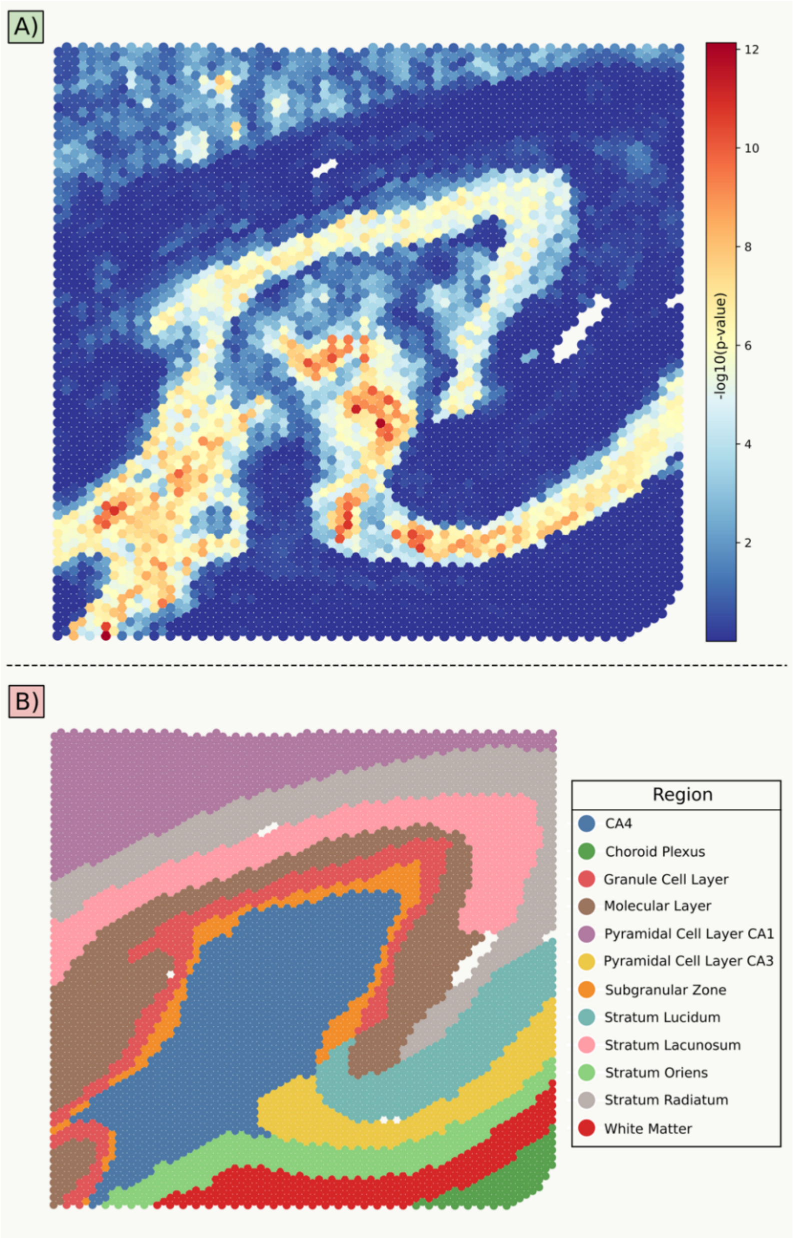

Supplementary Figure S1. Example of spot-level gsMap enrichments of schizophrenia common variant liability in a single capture area of the hippocampus A) gsMap (Song et al, 2025) heatmap showing enrichment Cauchy -$\log_{10}$ *p*-values for schizophrenia common variant association in spatial transcriptomic data of the hippocampus (Thompson et al, 2025) B) Annotation of hippocampal subregions from the same capture area, as determined by Thompson et al.

References

Song L, et al (2025): Spatially resolved mapping of cells associated with human complex traits. *Nature* 641: 932-941.

Thompson JR, *et al.* (2025): An integrated single-nucleus and spatial transcriptomics atlas reveals the molecular landscape of the human hippocampus. *Nature Neuroscience* 28: 1990–2004.

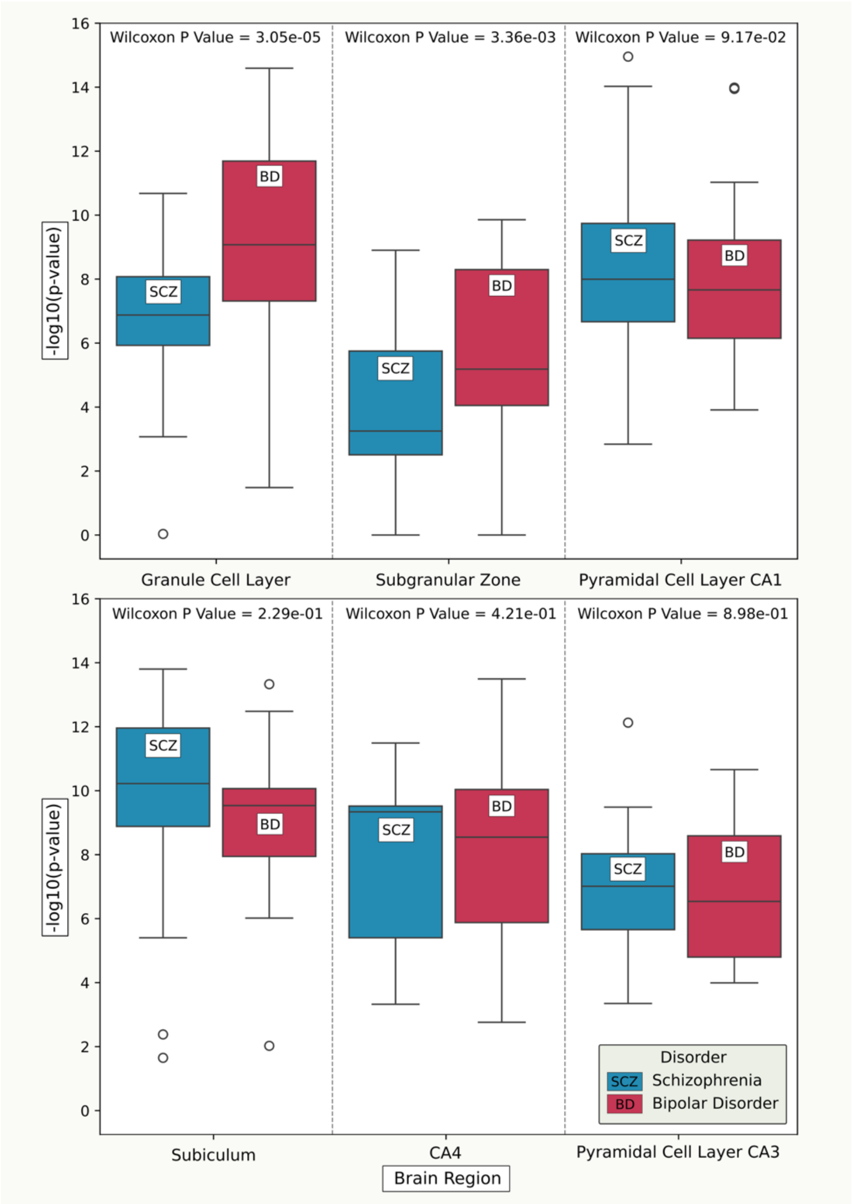

Supplementary Figure S2. Comparison between *p*-values for enrichment of bipolar disorder and schizophrenia common variant genetic risk in genes with high expression specificity for 6 subregions of the hippocampus enriched for both disorders. Boxplots show the median and interquartile range of -log10 Cauchy combination *p*-values for each disorder for all capture regions from each hippocampal subregion (between 9 and 16 capture regions per subregion). Whiskers extend to 1.5X the interquartile range beyond Q1 and Q3; circles show outlier *p*-values. Wilcoxon tests indicate significant differences in capture region Cauchy *p*-values between schizophrenia and bipolar disorder in the granule cell layer and subgranular zone of the dentate gyrus.

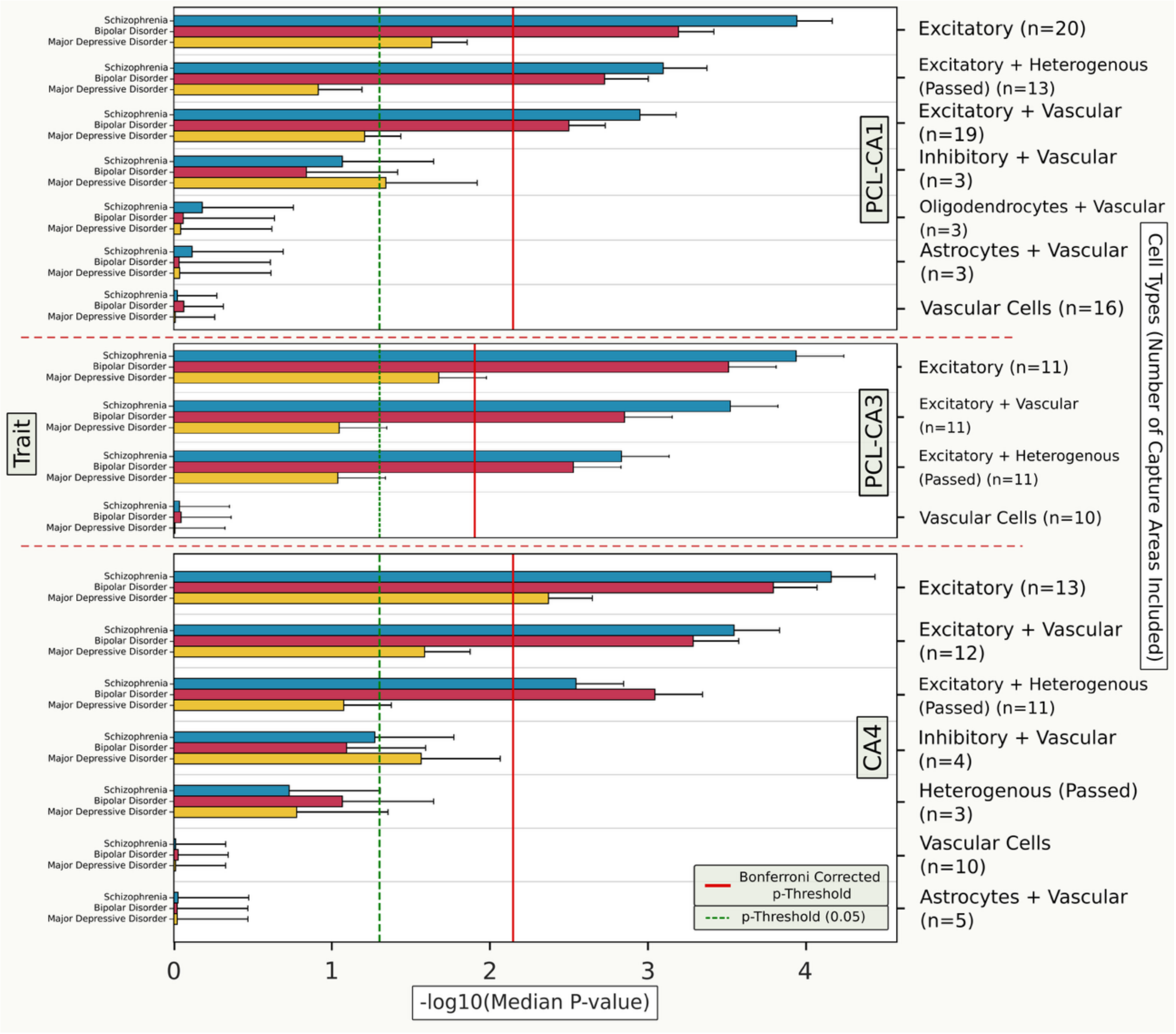

Supplementary Figure S3. **Enrichment of common variant genetic liability for schizophrenia, bipolar disorder and major depressive disorder in genes with high expression specificity for cell types of the CA1, CA3 and CA4 hippocampal subfields.** Bars indicate the -$\log_{10}$ median of the Cauchy *p*-values across capture areas for enrichment of trait associations for each cell type within each subfield. Error bars indicate standard errors of *p*-values across capture areas for each cell type. The number of capture areas for each subfield that included spots with the predicted cell type(s) are provided in parentheses. The solid red line indicates the Bonferroni *p*-value threshold for the number of cell types tested in each subfield. Note that the CA2 subfield was not distinguished in the original ST data.

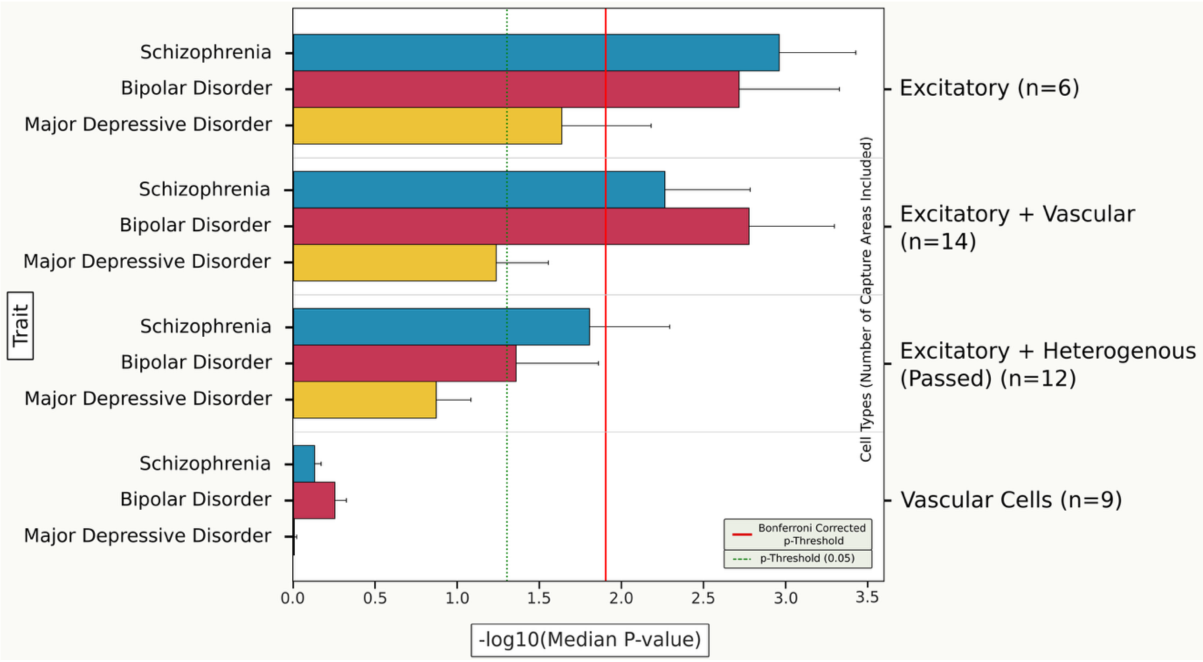

Supplementary Figure S4. **Enrichment of common variant genetic liability for schizophrenia, bipolar disorder and major depressive disorder in genes with high expression specificity for cell types of the dentate gyrus.** To maximize the number of spots containing each cell type, we combined spots belonging to the granule cell layer and subgranular zone. Bars indicate the -$\log_{10}$ median of the Cauchy *p*-values across capture areas for enrichment of trait associations for each cell type. Error bars indicate standard errors of *p*-values across capture areas for each cell type. The number of capture areas that included spots with the predicted cell type(s) are provided in parentheses. The solid red line indicates the Bonferroni *p*-value threshold for the number of cell types tested.

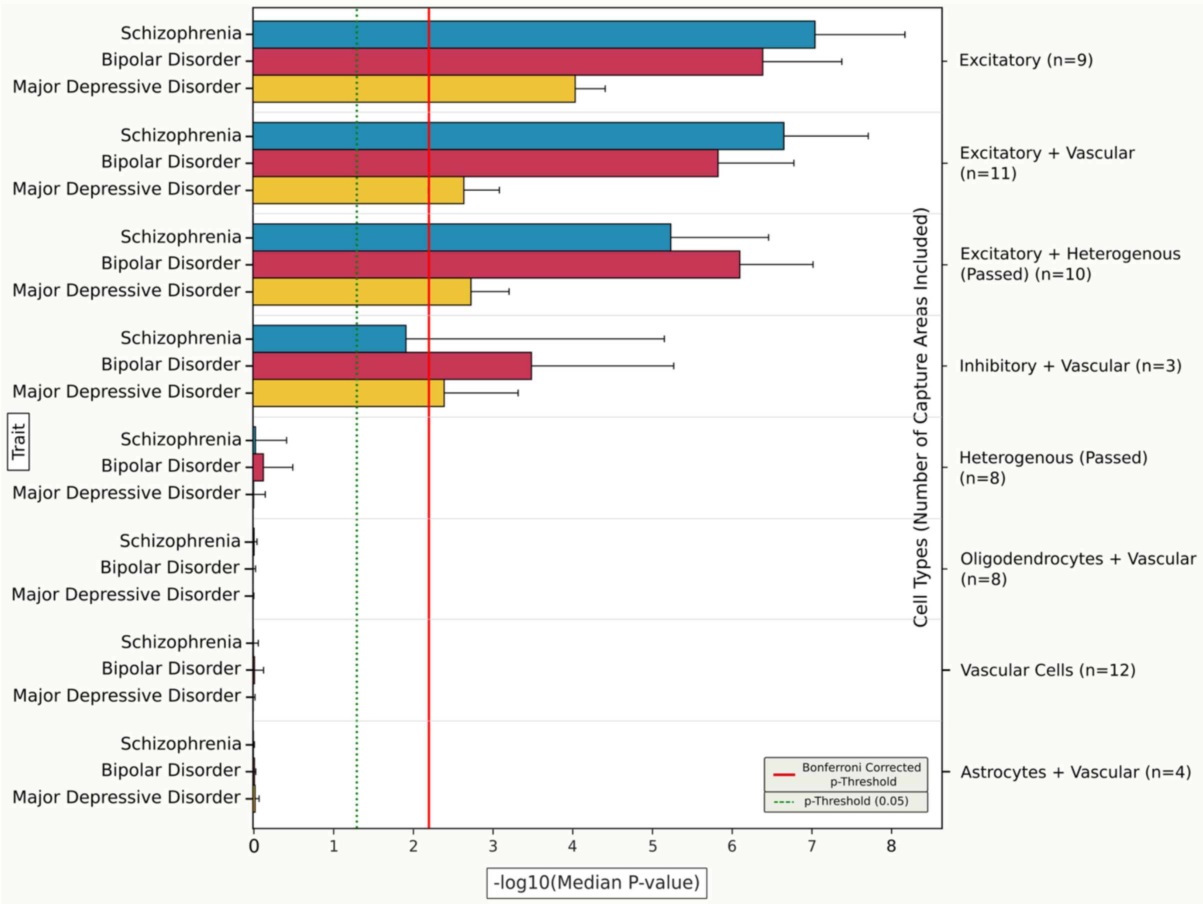

Supplementary Figure S5. Enrichment of common variant genetic liability for schizophrenia, bipolar disorder and major depressive disorder in genes with high expression specificity for cell types of the subiculum. Bars indicate the $-\log_{10}$ median of the Cauchy *p*-values across capture areas for enrichment of trait associations for each cell type. Error bars indicate standard errors of *p*-values across capture areas for each cell type. The number of capture areas that included spots with the predicted cell type(s) are provided in parentheses. The solid red line indicates the Bonferroni *p*-value threshold for the number of cell types tested.

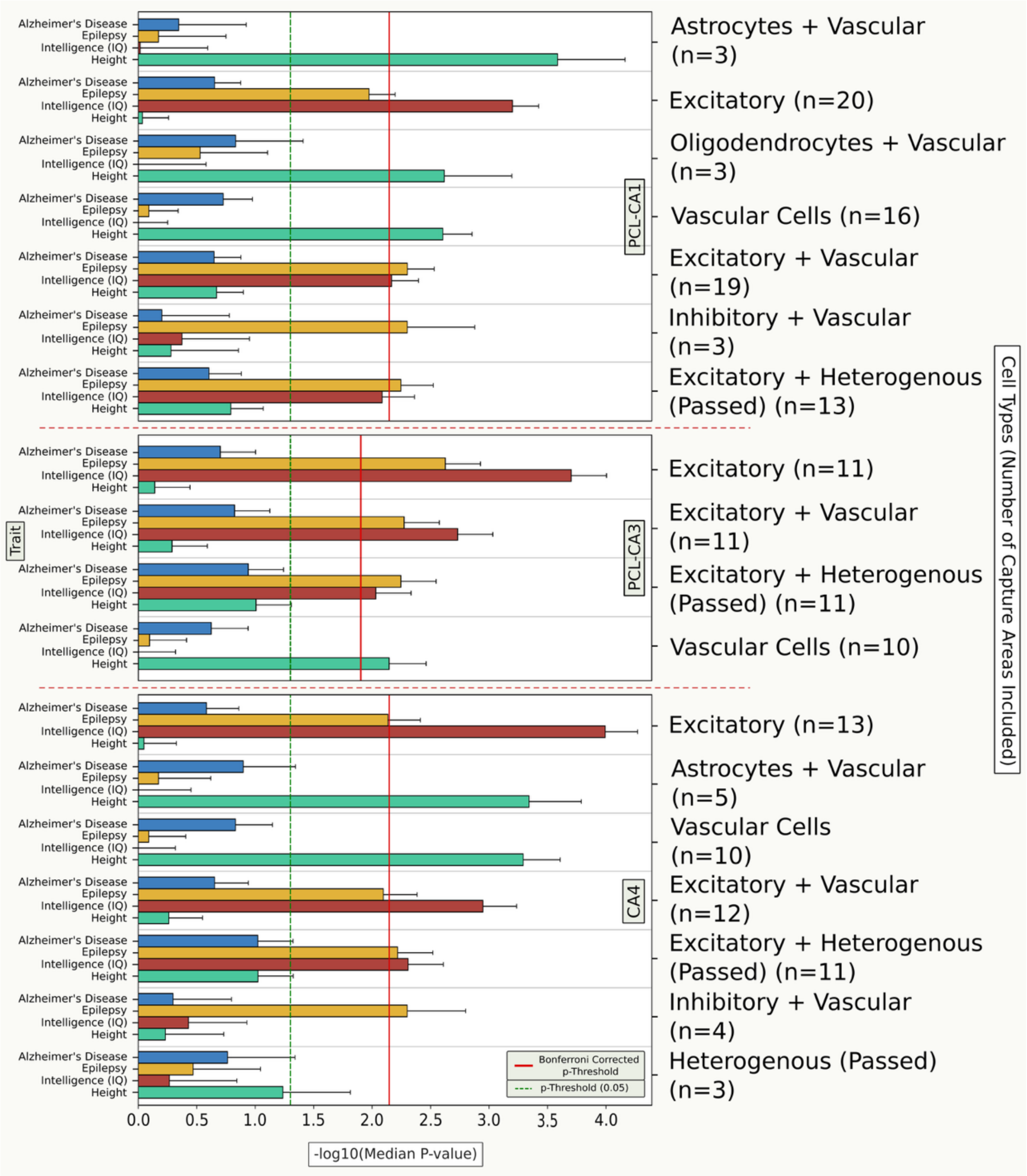

Supplementary Figure S6. Enrichment of common variants associated with Alzheimer's disease, genetic generalized epilepsy, intelligence and height in genes with high expression specificity for cell types of the CA1, CA3 and CA4 hippocampal subfields. Bars indicate the -$\log_{10}$ median of the Cauchy *p*-values across capture areas for enrichment of trait associations for each cell type within each subfield. Error bars indicate standard errors of *p*-values across capture areas for each cell type. The number of capture areas for each subfield that included spots with the predicted cell type(s) are provided in parentheses. The solid red line indicates the Bonferroni *p*-value threshold for the number of cell types tested in each subfield. Note that the CA2 subfield was not distinguished in the original ST data.

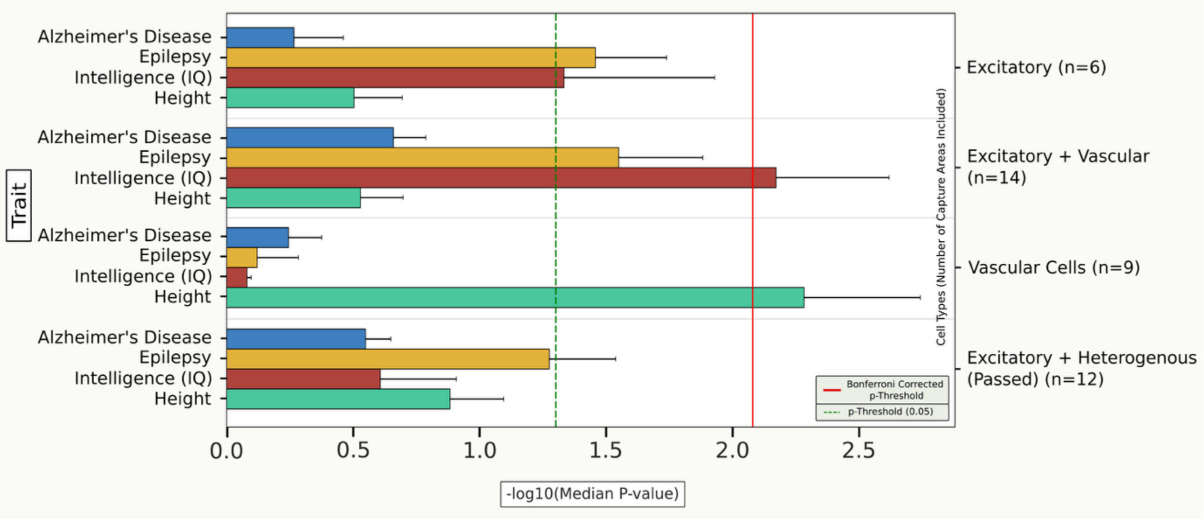

Supplementary Figure S7. Enrichment of common variants associated with Alzheimer's disease, genetic generalized epilepsy, intelligence and height in genes with high expression specificity for cell types of the dentate gyrus. To maximize the number of spots containing each cell type, we combined spots belonging to the granule cell layer and subgranular zone. Bars indicate the -$\log_{10}$ median of the Cauchy *p*-values across capture areas for enrichment of trait associations for each cell type. Error bars indicate standard errors of *p*-values across capture areas for each cell type. The number of capture areas that included spots with the predicted cell type(s) are provided in parentheses. The solid red line indicates the Bonferroni *p*-value threshold for the number of cell types tested.

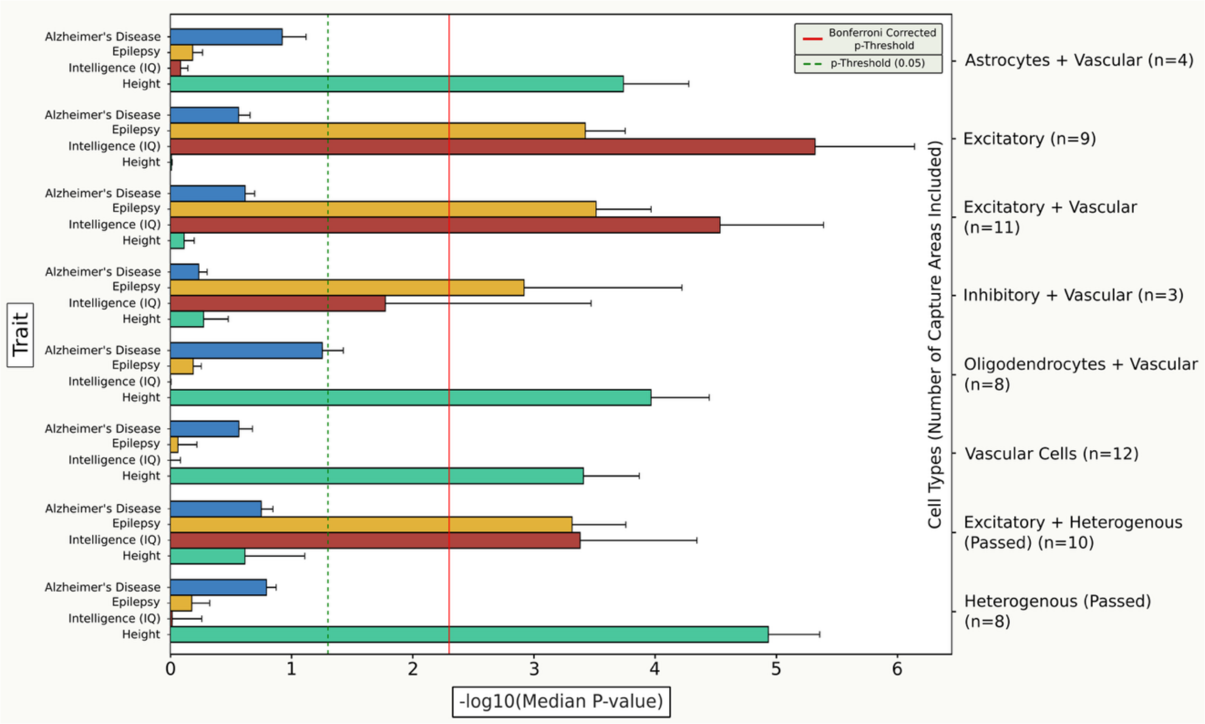

Supplementary Figure S8. Enrichment of common variants associated with Alzheimer's disease, genetic generalized epilepsy, intelligence and height in genes with high expression specificity for cell types of the subiculum. Bars indicate the -$\log_{10}$ median of the Cauchy *p*-values across capture areas for enrichment of trait associations for each cell type. Error bars indicate standard errors of *p*-values across capture areas for each cell type. The number of capture areas that included spots with the predicted cell type(s) are provided in parentheses. The solid red line indicates the Bonferroni *p*-value threshold for the number of cell types tested.
